# Rifaximin Reduces Risk of All-Cause Hospitalization in Cirrhotic Liver Transplant Candidates with Hepatic Encephalopathy

**DOI:** 10.3390/jcm12216871

**Published:** 2023-10-31

**Authors:** Simona Parisse, Quirino Lai, Francesca Martini, Alice Martini, Flaminia Ferri, Monica Mischitelli, Fabio Melandro, Gianluca Mennini, Massimo Rossi, Domenico Alvaro, Stefano Ginanni Corradini

**Affiliations:** 1 Department of Translational and Precision Medicine, “Sapienza” University of Rome, Viale dell’Università 37, 00185 Rome, Italy; simona.parisse@uniroma1.it (S.P.); francesca.martini@student.unisi.it (F.M.); a.martini23@student.unisi.it (A.M.); flaminia.ferri@uniroma1.it (F.F.); monica.mischitelli@uniroma1.it (M.M.); domenico.alvaro@uniroma1.it (D.A.); 2 General Surgery and Organ Transplantation Unit, “Sapienza” University of Rome, Viale del Policlinico 155, 00161 Rome, Italy; quirino.lai@uniroma1.it (Q.L.); fabio.melandro@uniroma1.it (F.M.); gianluca.mennini@uniroma1.it (G.M.); massimo.rossi@uniroma1.it (M.R.)

**Keywords:** rifaximin, hospitalization, hepatic-encephalopathy, liver transplant, cirrhosis

## Abstract

In cirrhotic patients listed for liver transplantation (LT) with a history of hepatic encephalopathy (HE), rifaximin reduces the number of hospitalizations, but whether it influences the time to first hospitalization is unknown. Aims: to evaluate the time-dependent impact of rifaximin on the risk of all-cause hospitalization and dropout in patients on the LT waiting list. Methods: Consecutive patients listed for LT were retrospectively enrolled. After balancing populations with and without rifaximin treatment using the inverse probability therapy weighting analysis, Fine–Gray multivariable competing risk analyses were run to explore risk factors for the first episode of hospitalization and dropout. Results: When comparing 92 patients taking rifaximin to the untreated group of 152, rifaximin treatment was not associated with any of the study outcomes. In the subset of patients with a history of HE at waitlist entry (N = 81 rifaximin-treated and N = 39 untreated), rifaximin intake was independently associated with a lower risk of hospitalization for all causes (SHR 0.638; 95.0% CI 0.418–0.973; *p* = 0.037) and for HE (SHR 0.379; 95.0% CI 0.207–0.693; *p* = 0.002). Conclusions: cirrhotic LT candidates with a prior history of HE rifaximin treatment are associated with a lower risk of time-dependent all-cause hospitalization, likely due to its unique effect on gut microbiome composition/function.

## 1. Introduction

Cirrhosis caused 1.32 million deaths worldwide in 2017 and, in 2019, cirrhosis mortality was associated with 2.4% of global deaths [1,2]. Rifaximin is a virtually non-absorbed oral antibiotic with antimicrobial activity against both aerobic and anaerobic Gram-positive and Gram-negative intestinal bacteria [3]. The efficacy of rifaximin in the prevention of recurrent hepatic encephalopathy (HE) and related hospital admissions has been widely demonstrated [4,5,6,7]. It has also been suggested that rifaximin may have a therapeutic effect beyond the treatment of HE in cirrhotic patients. In particular, a possible role of rifaximin has been hypothesized in improving systemic hemodynamics [8] and patient survival [9,10], reducing portal hypertension and its complications [8,10,11,12,13,14], and reducing the risk of infections [15,16] and hospitalizations [4,12,17,18,19]. However, the published data are contradictory on whether rifaximin can prevent complications of cirrhosis different from HE, related hospitalizations, and patient survival [5,8,14,16,20]. In theory, the best way to answer the question of whether rifaximin administration can reduce not only the risk of recurrence of HE but also the outcome of cirrhotic patients in general would be through prospective clinical trials with large numbers of patients [5]. However, currently, given the demonstrated and consolidated efficacy of rifaximin for the secondary prophylaxis of HE episodes, it appears ethically difficult to design new placebo-controlled clinical trials in patients who have experienced more than one HE episode [6]. This is even more true for patients on the liver transplant (LT) waiting list who are characterized by a high MELD score and are very frail. In fact, although LT represents the best treatment for decompensated cirrhosis, not all cirrhotic patients on the waiting list reach LT, due to complications of cirrhosis, including HE, most of which require hospitalization [21,22,23]. However, a negative effect of hospitalizations for HE on the survival of cirrhotic patients both before and after LT has been demonstrated [24,25,26,27,28,29,30]. In this retrospective study, we analyzed the impact of rifaximin treatment on the risk of first hospitalization, and on dropout for worsening or death in patients on the LT waiting list. We also performed a sub-analysis restricted to patients who had a history of HE at enrollment. 

## 2. Materials and Methods

### 2.1. Study Design

This was a retrospective monocenter observational study investigating data of cirrhotic patients listed for LT who received rifaximin or did not. The present study was conducted in accordance with the Declaration of Helsinki and was approved by the Local Ethics Board of Sapienza University of Rome (Ref. N. 3420. 27 November 2014). The Strengthening the Reporting of Observational Studies in Epidemiology (STROBE) guidelines were followed to create this study.

### 2.2. Study Population

Clinical records of 244 consecutive cirrhotic patients at the time of entry on the waiting list for LT at Sapienza University of Rome were retrospectively selected and evaluated (time period 2011–2018). Patients were divided into two groups based on whether they were on chronic treatment with rifaximin or not. All patients were ≥18 years old and cirrhosis diagnosis was made based on the presence of at least 2 of the following conditions: (a) history of cirrhosis complications: HE, variceal gastrointestinal bleeding, ascites; (b) blood test consistent with cirrhosis: hyperbilirubinemia, hypoalbuminemia, prolonged international normalized ratio, low platelet count; (c) signs of advanced chronic liver disease and/or portal hypertension at diagnostic examinations: nodular-appearing liver at abdominal imaging (ultrasound/computed tomography), reduced portal vein flow at ultrasound, increased liver stiffness, gastroesophageal varices at upper endoscopy; (d) fibrosis stage 4 according to Metavir classification at liver biopsy [31]. Exclusion criteria were absence of information on the assumption of rifaximin at the time of waiting list inscription; any concomitant bowel disease (e.g., celiac or inflammatory bowel disease); previous intestinal surgery (e.g., bowel resection); use of anti-inflammatory or probiotic drug in the six months before recruitment. 

### 2.3. Outcomes

The primary outcome of this study was the risk of first-episode hospitalization, either for all causes or for HE. Secondary outcomes were the risk of dropout for deterioration and for first episode of gastrointestinal (GI) bleeding and of spontaneous bacterial peritonitis (SBP). Dropout was considered an event in cases of (a) death; (b) clinical worsening; (c) tumor progression. In the cases of delisting for liver function improvement, change of LT center, and poor compliance, the cases were censored. The first episode of GI bleeding and that of SBP could be the cause of hospitalization or arise during hospitalization. Competitive risk analyses were performed for all outcomes, considering LT as the competitive event. 

### 2.4. Data Collection

Data were retrospectively extracted from hospitalization and outpatient follow-up records. Data errors and missingness were identified across the database and solved, when possible, with specific queries. Baseline information was collected at the time of the waitlist and included patient demographics, comorbidities, and cirrhosis etiology, including Metabolic-Associated Fatty Liver Disease (MAFLD) [32] and rifaximin treatment. To be diagnosed with MAFLD, as reported in a previous study, at least one of the following criteria had to be present: (1) body mass index (BMI) ≥ 25 kg/m^2^; (2) Type 2 Diabetes Mellitus (T2DM); (3) metabolic dysregulation, established by the presence of at least two of the following characteristics: (a) triglycerides ≥ 150 mg/dL or being on treatment for hypertriglyceridemia; (b) fasting serum glucose value of 100–125 mg/dL; (c) hypertension with median arterial blood pressure values ≥ 130/85 mmHg or being on treatment with antihypertensive drugs; (d) high-density lipoprotein cholesterol (HDL) less than 40 mg/dL in men or less than 50 mg/dL in women or being on treatment for low HDL [33]. Stage of liver disease and its complications were also recorded. Rifaximin was administered following the prescription policy of the respective waiting list period for each patient, taking into account the publication period of the guidelines [34]. The possible indications for the administration of rifaximin were history of overt HE or diagnosis of minimal HE in subjects who needed to drive. For the diagnosis of HE, the presence of at least one episode of overt HE prior to or at the time of waitlist entry was established according to the West-Haven criteria [6]. From the time of entry on the waiting list to the end of the follow-up, the number of episodes of GI bleeding and SBP and the number of hospitalizations for overt HE and for all causes were collected. Furthermore, the date of the first episode of GI and SBP and of the first hospitalization for HE and for all causes was recorded.

### 2.5. Statistical Analysis

Continuous variables are reported as medians and interquartile ranges (IQRs). Categorical variables are described as numbers and percentages. The Mann–Whitney U test and Fisher’s exact test compared continuous and categorical variables, respectively.

Missing data relative to study covariates always involved less than 10% of patients. In all the cases, missing data were handled with a single imputation method. In detail, a median of nearby points imputation was adopted. The median instead of the mean was adopted due to the skewed distribution of the managed variables.

To compensate for the nonrandomized design of this study, we balanced (or corrected for potential confounders) the populations using the inverse probability therapy weighting (IPTW) analysis. To compare the rifaximin group with the no-rifaximin group, we express continuous data as means (SDs) based on categorical data on the frequency distribution. Eight potential confounders were included in the boosted models: age, male sex, waiting time duration, Model for End-stage Liver Disease (MELD), BMI, T2DM, chronic kidney disease (CKD), and MAFLD. To reduce the artificial increase in the sample size, and therefore the type I error rate (i.e., increased number of false positives) associated with the inflated sample size in the pseudo-data, we used stabilized weights (SWs) according to the following formula:

SW = *p*/PS for the rifaximin group,

and SW = (1 − *p*)/(1 − PS) for the no rifaximin group,

where *p* is the probability of cause without considering covariates, and PS is the propensity score.

Because *p* values can be biased from population size, results from the comparisons between covariate subgroups are reported as effect size (Cohen d value). The Cohen d values that were lower than 0.1 indicated very small differences between means, values between 0.1 and 0.3 indicated small differences, values between 0.3 and 0.5 indicated moderate differences, and values greater than 0.5 indicated large differences.

Different Fine–Gray multivariable competing risk analyses were run in the post-IPTW population to explore the risk factors for all-cause hospitalization, GI bleeding, SBP, and dropout. The variables to use for constructing the models were preliminarily selected using Least Absolute Shrinkage and Selection Operator (LASSO) regression (stepwise regression with backward elimination), with the intent to create a parsimonious model in terms of number of covariates. Only variables present at the time of waiting list inscription were introduced in the models with the intent to avoid the risk of immortal time bias. The variables tested for each model were male sex, BMI, CKD, MELD, T2DM, age, cirrhosis etiology (HCV, HBV, alcohol, cryptogenic, other liver disease), HCC, ascites, and varices. The models were constructed using “liver transplantation” as the competing event. Sub-hazard ratios (SHRs) and 95.0% CIs were reported for significant variables. A sub-analysis only focusing on patients with HE at the time of waiting list inscription was also performed. 

Variables with *p* < 0.05 were considered statistically significant. Statistical analyses were run using the SPSS statistical package version 27.0 (SPSS Inc., Chicago, IL, USA). 

## 3. Results

Ninety-two patients were enrolled in the rifaximin group and one hundred fifty-two patients were enrolled in the no-rifaximin group. The median follow-up time was 239 days (IQR = 83–500). At enrollment, patients in the rifaximin group had been on rifaximin treatment for a median time of 145.5 days (IQR = 93.75–357) and the minimum treatment duration was 31 days. Table 1 shows the baseline characteristics of the investigated cohort. Patients in the rifaximin group had higher median values of MELD (*p* = 0.007) and MELDNa (*p* < 0.0001) and were more commonly affected by ascites (*p* = 0.02), esophageal varices (*p* = 0.04), and overt HE (*p* < 0.0001). In the rifaximin group, 12% of patients had no history of overt HE. In these patients, rifaximin was prescribed for a diagnosis of minimal HE. In patients in the rifaximin group, the diagnosis of T2DM (*p* = 0.02) and MAFLD (*p* = 0.004) was more frequent. 

During the follow-up, no differences were observed between the two groups in terms of dropout rates for worsening or LT (Table 2). From waitlisting to the end of the follow-up, as shown in Table 2, the median days to first hospitalization and the number of hospitalizations for all causes and HE, and the number of episodes of GI bleeding and SBP did not differ between the two groups. Similarly, the proportion of patients with at least one hospitalization for all causes and for HE, GI, and SBP did not differ between the two groups. 

### 3.1. Inverse Probability Therapy Weighting

With the intent to minimize the potential biases connected to the presence of confounders, the rifaximin-treated and untreated groups were artificially balanced using an IPTW. Before the balancing, the two groups showed small to moderate differences in the potential confounders investigated. After the IPTW, all the Cohen’s D-values declined, showing only very small differences after the balancing (Supplementary Table S1). 

### 3.2. Risk Factors for Hospitalization, GI Bleeding, SBP, and Dropout in the Entire Study Population after IPTW

Risk factors for the first episode of hospitalization for all causes or for HE, for the first episode of GI bleeding and of SBP, and for dropout were studied using a competitive risk analysis in the post-IPTW population (Table 3); rifaximin treatment was not statistically relevant in preventing these risks. For the risk of all-cause hospitalization or HE-related hospitalization, BMI, the presence of diabetes, and most importantly, MELD score were significant risk factors. BMI and MELD were risk factors for GI bleeding, while male gender was the only risk factor for SBP. In the case of dropout, alcoholic-related cirrhosis and BMI were risk factors, while CKD was protective, probably due to its weight in the MELD computation. 

### 3.3. Sub-Analysis in Patient with Hepatic Encephalopathy at Waiting List Inscription

The long recruitment period (2011–2018) allowed us to carry out a sub-analysis in the subgroup of patients with HE by dividing them into treated and not treated with rifaximin. In this sub-analysis, 36 patients were not on rifaximin treatment, while 81 were on rifaximin treatment. This difference in treatment choice was due to the different time period of inscription on the waiting list compared to the date of publication of the recommendation on the management of HE and the related prescriptive policy of rifaximin. Both before and after IPTW, no significant differences were observed between the rifaximin and untreated groups in terms of demographic and clinical characteristics (Supplementary Table S2). After IPTW, 81 rifaximin-treated patients were compared with 39 untreated patients and the two groups were more balanced in terms of CKD than before IPTW. During follow-up, no differences were observed between the two groups in terms of dropout rates for worsening or LT (Table 4). From the waitlist to the end of follow-up, as shown in Table 4, the proportion of patients with at least one HE hospitalization and the median number of HE hospitalizations were lower in the rifaximin group than in the untreated group. Although there were no significant intergroup differences in the proportion of patients with at least one hospitalization for all causes and the median number of hospitalizations for all causes, the rifaximin group had a median number of days elapsed before first hospitalization for all causes greater than the untreated group. The number of episodes of GI bleeding and SBP and the percentage of patients with at least one episode of GI bleeding and SBP did not differ between the two groups. 

Interestingly, in the multivariate analysis post IPTW, rifaximin treatment was found to be a protective factor both for the risk of all-cause hospitalization, with SHR = 0.64 (*p* = 0.04), and for the risk of HE-related hospitalization (SHR = 0.38, *p* = 0.002) (Table 5). In contrast, rifaximin treatment failed to show a benefit on the risk of dropout and GI or SBP episodes. 

## 4. Discussion

We explored the potential clinical benefits of rifaximin treatment in cirrhotic patients entering the waiting list for LT.

In our retrospective cohort, more than one third (37.7%) of cirrhotic patients undergoing LT were on rifaximin treatment since entering the waiting list and most of them (88%) had a history of overt HE. MAFLD represents, as expected, the main etiology of liver cirrhosis and was more common in the rifaximin-treated group than in the untreated group. For a better comparison between the rifaximin-treated and untreated group and in order to compensate for differences due to the nonrandomized design of our study, we balanced the two populations using the IPTW analysis. This statistical method is considered a good tool to compare differences between groups for all baseline characteristics, both before and after confounder weighting, and to estimate the treatment effect in observational studies. Compared to other statistical methods, the main advantages of IPTW are that of increasing the effective size of the sample, retaining most of individuals in the analysis, and of showing less bias in the estimation of hazard ratios [35]. In our study, despite the more severe clinical conditions (see Table 1 and Table 2) of the rifaximin group, already reported in a previous study, the number of hospitalizations and dropouts during the waiting list period did not differ from the untreated group [36]. Although rifaximin treatment did not influence waiting list outcomes, we found its protective role in the subgroup of patients who had a history of overt HE at the time of listing. In detail, in this subset of patients, rifaximin had a significant impact not only, as expected, on the risk of hospitalization related to HE, but also on hospitalization for all causes. In our study, we collected data regarding the time elapsed between entry into the waiting list for LT and the first hospitalization for any cause. Unfortunately, we do not have a reason for all first hospitalizations (i.e., acute kidney injury, infections, bleeding/anemization, electrolyte imbalances, etc.). Furthermore, despite having collected the episodes of GI bleeding and SBP, we cannot say when they constituted the reason for hospitalization.

The benefit of rifaximin on all-cause hospitalization in patients with advanced cirrhosis such as LT candidates and with a prior history of HE is relevant for several reasons. Indeed, for patients on the LT waiting list, avoiding hospitalization improves the chances of accessing the transplant, as hospitalization in these patients has been associated with worst outcomes. In fact, patients who are hospitalized often have a temporary or definitive contraindication to LT due to the complication/s or nosocomial infections that delay or prevent access to the transplant [21]. Our results are consistent with a previous study, conducted outside the transplant field, where rifaximin in advanced cirrhotic patients delays the time to first hospitalization for all causes. It is interesting that in this study, the beneficial effect of rifaximin was limited to patients with advanced disease (MELD score ≥ 12) [11]. Our results are also in keeping with Salehi et al. who demonstrated that treatment with rifaximin had a protective effect on the number of hospitalizations for all-causes, for SBP, for ascites, and for variceal bleeding [12].

Our result that rifaximin is of benefit only in the subgroup of patients with a prior history of HE deserves specific comments. It has been shown, in fact, that the gut microbiota characteristics are different in patients with and without a history of HE and it is likely that the effects of rifaximin may differ in the two types of patients [37,38]. Indeed, the effect of rifaximin administration on gut microbiota-derived inflammation has been demonstrated to be relevant in patients with a history of overt HE [8,14,16,20,39]. Studies aimed at verifying any beneficial effects of rifaximin on the outcomes of cirrhotic patients other than HE should therefore provide for a balance, in the group treated with rifaximin compared to the untreated group, of patients with or without a previous history of HE. In this regard, in the past, the administration of rifaximin was beneficial on some outcomes of cirrhotic patients other than HE in some studies in which the percentage of patients with or without a previous history of HE was not specified for both groups, the one treated with rifaximin and the untreated one [9,13,40,41], or in two studies in which the percentage of patients with or without a history of HE was balanced in the two study groups [42,43]. 

Limitations of our study are the monocentric and retrospective nature. This study was observational in a heterogeneous population and not rigorously designed to study the effect of treatment with rifaximin versus no rifaximin. Especially regarding the analysis in the entire study population, the rifaximin-treated and untreated groups were unbalanced in terms of disease severity, comorbidity, and etiology. It is possible that this imbalance was not completely corrected by the statistical analysis with IPTW and by the fact of having tested, as potential confounders for the multivariate analyses, some disease severity indices such as the MELD score; the presence of HCC, ascites, and varices; and different demographic variables and comorbidities. Furthermore, we did not collect data regarding GI bleeding and SBP prophylaxis, which may have influenced the results. Finally, we cannot say what, in addition to HE, are the other types of complications that cause hospital admissions for all causes on which Rifaximin has a beneficial effect in the subgroup with previous HE. However, the strengths of our study are the long recruitment period (ten years), during which all cirrhotic patients underwent the same waitlist and dropout criteria, and the long median follow-up (>7.5 months).

In summary, our study found that a large percentage of cirrhotic patients on the waiting list for LT are treated with rifaximin and that, among the latter, MAFLD is the prevalent etiology. Our study demonstrated that, in cirrhotic patients with a history of established HE at the time of the liver transplant waiting list, rifaximin has a significant beneficial impact on the risk of hospitalization related to HE and all causes. 

## Figures and Tables

**Table 1:** Baseline characteristics at liver transplantation waitlist entry of patients in the entire cohort divided into rifaximin-treated and untreated groups.

Variable	Rifaximin *n* = 92 (37.7%)	No Rifaximin *n* = 152 (62.3%)	*p*-Value
	Median (IQR) or *n* (%)		
Age, years	58 (53–62)	57 (49–62)	0.19
Male sex	81 (88.0)	118 (77.6)	0.06
MELD MELDNa	15 (13–19) 18 (16–22)	15 (10–18) 16 (11–20)	0.007 <0.0001
BMI	26.2 (24.4–28.8)	25.4 (23.2–27.8)	0.07
T2DM	35 (38.0)	36 (23.7)	0.02
CKD	6 (6.5)	21 (13.8)	0.09
HCC	36 (39.1)	64 (42.2)	0.69
Ascites Mild Moderate Severe	6 (6.5) 10 (10.9) 12 (13.0)	15 (9.9) 5 (3.3) 10 (6.6)	0.02
Varices F1 F2 F3	25 (27.2) 23 (14.1) 2 (2.2)	19 (12.5) 27 (17.8) 5 (3.3)	0.04
History of overt HE	81 (88.0)	36 (23.7)	<0.0001
Oral non-adsorbable disaccharides	84 (91.0)	38 (25.0)	<0.0001
Portal thrombosis	15 (16.3)	18 (11.8)	0.34
MAFLD Alcohol HCV HBV Cryptogenic Other	68 (73.9) 40 (43.5) 32 (34.8) 9 (9.8) 7 (7.6) 5 (5.4)	84 (55.3) 56 (36.8) 52 (34.2) 22 (14.5) 6 (3.9) 24 (15.8)	0.004 0.35 1.00 0.33 0.25 0.02

All data refer to baseline. Abbreviations: BMI, body mass index (kg/m^2^); CKD, chronic kidney disease; HBV, hepatitis B virus; HCC, hepatocellular carcinoma; HCV, hepatitis C virus; HE, hepatic encephalopathy; IQR, interquartile range; MAFLD, Metabolic-Associated Fatty Liver Disease; MELD, Model for End-stage Liver Disease; MELDNa, Model for End-stage Liver Disease Sodium; T2DM, type 2 diabetes mellitus.

**Table 2:** Events recorded during the follow-up among the entire cohort divided into rifaximin-treated and untreated groups.

Variable	Rifaximin *n* = 92 (37.7%)	No Rifaximin *n* = 152 (62.3%)	*p*-Value
	Median (IQR) or *n* (%)		
Number of patients with at least one: All-cause hospitalization Hospitalization due to HE Episode of GI bleeding Episode of SBP	78 (84.8) 26 (28.3) 22 (23.9) 5 (5.4)	124 (81.6) 30 (19.7) 35 (23.0) 5 (3.3)	0.60 0.16 0.77 0.38
Number of: All-causes hospitalizations Hospitalizations due to HE Episodes of GI bleeding Episodes of SBP	2 (1–4) 0 (0–1) 0 (0–1) 0 (0)	2 (1–3) 0 (0) 0 (0) 0 (0)	0.20 0.13 0.51 0.51
LT	49 (53.3)	86 (56.6)	0.61
Dropout Death HCC progression Liver function worsening	31 (33.7) 22 (23.9) 5 (5.4) 4 (4.4)	46 (30.3) 28 (18.4) 9 (5.9) 9 (5.9)	0.87
Days to first hospitalization	59 (19.25–192)	40.5(12.25–93.25)	0.06

All data were recorded during the follow-up. Abbreviations: GI, gastro-intestinal; HCC, hepatocellular carcinoma; HE, hepatic encephalopathy; IQR, interquartile range; LT, liver transplant; SBP, spontaneous bacterial peritonitis.

**Table 3:** Competing-risk analysis in the entire study population after IPTW: risk factors for all-cause hospitalization, encephalopathy-related hospitalization, GI bleeding episode, spontaneous bacterial peritonitis episode, and dropout.

Variables	Beta	SE	SHR	95.0% CI		*p*-Value
				Lower	Upper	
All-cause hospitalization						
MELD	0.106	0.033	1.111	1.094	1.209	0.002
BMI	0.118	0.048	1.125	1.093	1.210	0.014
T2DM	0.833	0.369	2.301	2.107	2.439	0.024
Encephalopathy-related hospitalization						
MELD	0.104	0.033	1.110	1.093	1.210	0.002
BMI	0.120	0.046	1.128	1.101	1.214	0.009
T2DM	0.825	0.365	2.281	2.091	2.437	0.024
GI bleeding episode						
BMI	0.136	0.053	1.145	1.121	1.267	0.011
MELD	0.081	0.036	1.084	1.073	1.094	0.023
Spontaneous bacterial peritonitis episode						
Male sex	1.214	0.088	2.729	1.976	3.421	<0.001
Dropout						
Alcohol	0.745	0.243	2.107	1.785	2.379	0.002
BMI	0.093	0.038	1.098	1.087	1.123	0.015
CKD	−1.179	0.509	0.308	0.221	0.457	0.021

All data refer to baseline in the post-IPTW population. Abbreviations: BMI, body mass index (kg/m^2^); CI, Confidence Interval; CKD, chronic kidney disease; GI, gastro-intestinal; MELD, Model for End-stage Liver Disease; SE, standard error; SHR, sub-distribution hazard ratio; T2DM, type 2 diabetes mellitus.

**Table 4:** Events recorded during the follow-up among the sub-group of patients with a history of hepatic encephalopathy at the time of waiting list inscription (before and after IPTW population).

	Before-IPTW			After-IPTW		
Variable	Rifaximin *n* = 81	No Rifaximin *n* = 36	*p*-Value	Rifaximin *n* = 82	No Rifaximin *n* = 39	*p*-Value
	Median (IQR) or *n* (%)			Median (IQR) or *n* (%)		
Number of patients with at least one: All-cause hospitalization Hospitalization due to HE Episode of GI bleeding Episode of SBP	69(85.2) 25(30.9) 21(25.9) 5(6.1)	35(97) 21(58.3) 12(33.3) 3(8.3)	0.07 0.006 0.4 0.6	71(88.8) 25(30.9) 21(25.9) 5(6.3)	38(97.4) 23(59.0) 13(33.3) 4(10.3)	0.2 0.005 0.4 0.5
Number of: All-causes hospitalizations Hospitalizations due to HE Episodes of GI bleeding Episodes of SBP	2(1–4) 0(0–1) 0(0–1) 0(0)	3(2–4) 1(0–2) 0(0–1) 0(0)	0.1 0.006 0.5 0.7	2(1–4) 0(0–1) 0(0–1) 0(0)	3(2–4) 1(0–2) 0(0–1) 0(0)	0.2 0.003 0.5 0.6
LT	42(51.9)	21(58.3)	0.5	42(51.2)	23(59.0)	0.5
Dropout Death HCC progression Liver function worsening	21(25.9) 5(6.2) 3(3.7)	10(27.8) 0(0) 2(5.5)	0.3	23(28.0) 4(4.9) 3(3.7)	12(30.8) 0(-) 2(5.1)	0.4
Days to first hospitalization	69(21–204.5)	20.5(9.3–68.8)	0.01	67.1(23–199.7)	22.3(10.4–69.8)	0.02

All data were recorded from waitlisting to the end of follow-up. Abbreviations: GI, gastro-intestinal; HE, hepatic encephalopathy; IQR, interquartile range; LT, liver transplant; SBP, spontaneous bacterial peritonitis.

**Table 5:** Sub-analysis in patients with a prior history of HE at the time of waitlisting. Competing-risk analysis: risk factors for all-cause hospitalization, encephalopathy-related hospitalization, GI bleeding episode, spontaneous bacterial peritonitis episode, and dropout.

Variables	Beta	SE	SHR	95.0% CI		*p*-Value
				Lower	Upper	
All-cause hospitalization						
MELD	0.068	0.025	1.071	1.020	1.124	0.006
BMI	0.079	0.031	1.082	1.018	1.151	0.011
MAFLD	−0.618	0.256	0.539	0.326	0.891	0.016
Rifamixin	−0.450	0.216	0.638	0.418	0.973	0.037
Encephalopathy-related hospitalization						
Rifamixin	−0.971	0.308	0.379	0.207	0.693	0.002
MELD	0.106	0.035	1.112	1.039	1.190	0.002
WT duration	0.013	0.005	1.013	1.003	1.024	0.014
GI bleeding episode						
BMI	0.203	0.052	1.225	1.106	1.356	<0.0001
MAFLD	−1.137	0.469	0.321	0.128	0.804	0.015
MELD	0.077	0.046	1.080	0.987	1.181	0.092
Spontaneous bacterial peritonitis episode						
MELD	0.239	0.092	1.271	1.061	1.521	0.009
Age	−0.158	0.060	0.854	0.758	0.961	0.009
Dropout						
MAFLD	−0.991	0.407	0.371	0.167	0.824	0.015
MELD	0.097	0.041	1.102	1.016	1.195	0.018
BMI	0.119	0.052	1.126	1.016	1.248	0.023

Abbreviations: BMI, body mass index (kg/m^2^); CI, Confidence Interval; GI, gastro-intestinal; MAFLD, Metabolic-Associated Fatty Liver Disease; MELD, Model for End-stage Liver Disease; SE, standard error; SHR, sub-distribution hazard ratio; WT waiting time.

## Data Availability

Data are available upon request.

## Supplementary Materials

The following supporting information can be downloaded at https://www.mdpi.com/article/10.3390/jcm12216871/s1. Supplementary Table S1: Effect of stabilized IPTW in the entire study population on the variables used for balancing the two groups; Supplementary Table S2: Patient-related characteristics: sub-group of patients with a history of hepatic encephalopathy at the time of waiting list inscription before and after IPTW.
