# Supplementary material for: Rifaximin Reduces Risk of All-Cause Hospitalization in Cirrhotic Liver Transplant Candidates with Hepatic Encephalopathy
Source: J Clin Med. 2023 Oct 31;12(21):6871. doi: 10.3390/jcm12216871 (PMC10647372; doi:10.3390/jcm12216871)
Supplement: Supplementary file 1 [file jcm-12-06871-s001.zip › jcm-2680301-supplementary.pdf]

Journal of
Clinical Medicine

Supplementary Table S1. Effect of stabilized IPTW in the entire study population on the variables used for balancing the two groups

Variables	Pre-IPTW	Post-IPTW
	**Cohen's D-value**	**Cohen's D-value**
Age, years	0.24	-0.02
Male sex	0.26	-0.02
Waiting time duration	-0.13	-0.08
MELD	0.36	-0.03
BMI	0.22	0.01
T2DM	0.31	-0.01
CKD	-0.22	-0.04
MAFLD	0.40	0.02

All data refer to baseline unless differently specified.

Cohen d values indicate the effect size. Values < 0.1 indicate very small effect size; values between 0.1 and < 0.3 small, values between > 0.3 and < 0.5 moderate, values > 0.5 large.

Abbreviations: BMI, body mass index (Kg/m^2); CKD, chronic kidney disease; IPTW, inverse probability therapy weighting; MAFLD, metabolic associated fatty liver disease; MELD, Model for End stage Liver Disease; T2DM, type 2 diabetes mellitus.

Supplementary Table S2. Patient-related characteristics: sub-group of patients with a history of hepatic encephalopathy at the time of waiting list inscription before and after IPTW.

	Before-IPTW			After-IPTW		
**Variable**	Rifaximin n=81	No rifaximin n=36	P-value	Rifaximin n=81	No rifaximin n=39	P-value
	Median (IQR) or n (%)			Median (IQR) or n (%)		
Age, years	57.7(52.9-62)	58(49.2-62.2)	0.33	57 (52-61)	58 (51-62)	0.78
Male sex	72(88.9)	30(83.3)	0.40	68 (84.0)	33 (84.6)	1.00
MELD	15.8(13.2-19.3)	16.3(12-19.8)	0.79	15 (13-18)	16 (13-21)	0.32
MELDNa	18(15-22.1)	18.6(13-22)	0.53	17 (14-21)	18 (14-22)	0.75
BMI	25.9(24.5-29)	26.7(23.3-29.9)	0.97	25.9 (24.1-28.5)	26.5 (23.5-28.7)	0.84
Comorbidity						
T2DM	33(40.7)	14(38.9)	0.81	25 (30.9)	17 (43.6)	0.22
CKD	5(61.2)	6(16.7)	0.08	11 (13.6)	5 (12.8)	1.00
HCC	32(39.5)	14(38.9)	0.75	29 (35.8)	15 (38.5)	0.84
Ascites			0.42			0.12
Mild	3(3.7)	4(11.1)		3 (3.7)	4 (10.3)	
Moderate	1(1.2)	9(25)		12 (14.8)	1 (2.6)	
Severe	4(4.9)	11(30.5)		12 (14.8)	5 (12.8)	
Varices			0.53			0.79
F1	20(24.7)	6(16.7)		19 (23.5)	7 (17.9)	
F2	12(14.8)	9(25)		13 (16.0)	9 (23.1)	
F3	2(2.5)	1(2.8)		2 (2.5)	1 (2.6)	
Portal thrombosis	11(13.6)	3(8.3)	0.4	12 (14.8)	3 (7.7)	0.38
MAFLD	63(77.8)	24(66.7)	0.69	53 (65.4)	27 (69.2)	0.84
Alcohol	37(45.7)	15(41.6)	0.52	37 (45.7)	17 (43.6)	0.85
HCV	27(33.3)	12(33.3)	0.86	12 (30.8)	28 (34.6)	0.84
HBV	6(7.4)	7(19.4)	0.06	5 (6.3)	7 (17.9)	0.06
Cryptogenic	6(7.4)	3(8.3)	0.91	6 (7.4)	3 (7.7)	1.00
Other	4(4.9)	2(5.6)	0.94	5 (6.2)	1 (2.6)	0.66

Abbreviations: BMI, body mass index (Kg/m^2); CKD, chronic kidney disease; GI, gastro-intestinal; HBV hepatitis B virus; HCV, hepatitis C virus; HE, hepatic encephalopathy; HCC, hepatocellular carcinoma; IQR, interquartile range; LT, liver transplantation; MAFLD, metabolic associated fatty liver disease; MELD, Model for End stage Liver Disease; MELDNa, Model for End Stage Liver Disease Sodium; PBS, spontaneous bacterial peritonitis; T2DM, type 2 diabetes mellitus.
